# Duration of depressive symptoms and mortality risk: the English Longitudinal Study of Ageing (ELSA)

**DOI:** 10.1192/bjp.bp.114.155333

**Published:** 2016-04

**Authors:** James White, Paola Zaninotto, Kate Walters, Mika Kivimäki, Panayotes Demakakos, Jane Biddulph, Meena Kumari, Cesar De Oliveira, John Gallacher, G. David Batty

**Affiliations:** **James White**, PhD, School of Medicine, Cardiff University, Cardiff; **Paola Zaninotto**, PhD, **Kate Walters**, PhD, **Mika Kivimäki**, PhD, **Panayotes Demakakos**, PhD, **Jane Biddulph**, PhD, **Meena Kumari**, PhD, **Cesar De Oliveira**, PhD, Department of Epidemiology and Public Health, University College London, London; **John Gallacher**, PhD, Cochrane Institute of Primary Care and Public Health, School of Medicine, Cardiff University, Cardiff; **G. David Batty**, DSc, Department of Epidemiology and Public Health, University College London, London, Centre for Cognitive Ageing and Cognitive Epidemiology, University of Edinburgh, Edinburgh, Alzheimer Scotland Dementia Research Centre, University of Edinburgh, Edinburgh, UK

## Abstract

**Background**

The relationship between the duration of depressive symptoms and mortality remains poorly understood.

**Aims**

To examine whether the duration of depressive symptoms is associated with mortality risk.

**Method**

Data (*n* = 9560) came from the English Longitudinal Study of Ageing (ELSA). We assessed depressive symptom duration as the sum of examinations with an eight-item Center for Epidemiologic Studies Depression Scale score of ⩾3; we ascertained mortality from linking our data to a national register.

**Results**

Relative to those participants who never reported symptoms, the age- and gender-adjusted hazard ratios for elevated depressive symptoms over 1, 2, 3 and 4 examinations were 1.41 (95% CI 1.15–1.74), 1.80 (95% CI 1.44–2.26), 1.97 (95% CI 1.57–2.47) and 2.48 (95% CI 1.90–3.23), respectively (*P* for trend <0.001). This graded association can be explained largely by differences in physical activity, cognitive function, functional impairments and physical illness.

**Conclusions**

In this cohort of older adults, the duration of depressive symptoms was associated with mortality in a dose–response manner.

A series of cohort studies have documented that people with major depression or those reporting depressive symptoms are at an increased risk for mortality.^1–7^ These findings are, however, largely based on studies with a single assessment of depressive symptoms. As symptoms of depression tend to fluctuate over time,^1,2^ these studies may not have fully captured the impact of these symptoms on mortality risk. In the few cohort studies with repeated assessments of depressive symptoms, investigators have not directly examined the role of the duration of depressive symptoms on mortality; instead, they have examined the impact of incident,^3^ intermittent^4–6^ or chronic depression.^4–7^ In the only study to examine the duration of depressive symptoms, an increased risk of mortality was found when symptoms persisted for 3 years;^5^ however, the small number of cases (*n* = 226) and deaths (*n* = 79) in this cohort meant that analysis into the duration when mortality risk became raised was not undertaken. The English Longitudinal Study of Ageing (ELSA), with its multiple phases of data collection and large number of deaths, represents an unusual opportunity to explore the association between the duration of depressive symptoms and mortality.

## Method

### Sample

Established in 2002–2003, ELSA is a biannual, ongoing, nationally representative, prospective cohort study of health and ageing.^8^ The initial interview in 2002–2003 (wave 1) was based on 11 391 adults aged 50 or older who had earlier participated in the Health Survey for England (HSE).^9^ The second interview was held in 2004–2005 (wave 2), the third in 2006–2007 (wave 3) and the fourth in 2008–2009 (wave 4). The National Research Ethics Service (MREC/01/2/91) provided the ethical approval for ELSA.

### Measures

#### Depressive symptoms

We measured depressive symptoms with the eight-item version of the Center for Epidemiologic Studies Depression Scale (CES-D), a widely used self-report measure of depressive symptoms, used to identify people at risk of depression in population-based studies.^10^ Items capture information on symptoms of negative affect and somatic complaints experienced in the past week. A dichotomous (yes/no) response to each item resulted in a total score ranging between 0 (no symptoms) and 8 (all eight symptoms). This short version had good internal consistency at each wave (Cronbach's α>0.95) and comparable psychometric properties to the full 20-item CES-D.^10,11^ A total CES-D score of 3 or greater was used to denote ‘caseness’; this definition has been validated against standardised psychiatric interviews in older populations.^11^ To derive a duration of depression symptoms score, we totalled the number of occasions a person was recorded as being a case at each of the four waves, resulting in a score range from 0 (never a case) to 4 (a case at all four waves).

#### Confounding variables

We obtained information on social, demographic, clinical and lifestyle covariates from the wave 1 interview. Socioeconomic position was based on the National Statistics Socio-economic Classification (NS-SEC) and quintiles of total wealth, defined from the sum of financial, physical (e.g. businesses, land) and housing wealth, minus debts and pension payments.^12^ We defined cohabitation status as currently living alone or not. Self-reported health behaviours included smoking status (current; ex-smoker; never smoked), frequency of alcohol consumption in the past year (twice a day or more; daily or almost daily; once or twice a week; once or twice a month; special occasions only; not at all) and physical activity during leisure time, recorded as participation in vigorous, moderate and mild activities (more than once per week; once per week; 1–3 times per month; hardly ever).^13^ Measures of chronic disease included lifetime self-reported physician diagnoses of chronic conditions (chronic obstructive pulmonary disorder; asthma; diabetes; arthritis), circulatory disease (hypertension; prior heart attack; stroke; angina; heart conditions (murmur, abnormal heart rhythm, congestive heart failure)) and cancer. The use of antidepressants was recorded at wave 0 (HSE interviews in 1998, 1999 and 2001). We derived two measures of cognitive function from a set of tests administered during an interview. A memory test included questions on worry about memory, orientation in time, verbal learning and recall, and prospective memory.^14^ Executive function assessments included verbal fluency and letter cancellation tasks. We combined scores, with higher scores indicating better functioning.^14^ (A detailed description of these tests is provided in the online data supplement.)

We assessed impairments in physical function by asking participants whether they experienced difficulties, for at least 3 months, with six activities of daily living (ADLs) (e.g. bathing or showering, eating) and seven instrumental activities of daily living (IADLs) (e.g. taking medications, preparing a hot meal).^14^ As impairments in physical function were relatively rare, we classified participants reporting one or more problems with an ADL or IADL as having a functional impairment.

#### Ascertainment of mortality

We ascertained all-cause mortality for consenting study members (11 104; 97.5% of eligible participants) by linking our data to the UK National Health Service mortality registry up to March 2012.

### Statistical methods

To test differences in baseline characteristics by the duration of depressive symptoms, we used χ^2^ for categorical variables and analysis of variance for continuous variables. We ascertained that the proportional hazards assumption had not been violated by inspecting the log (−log(survival)) plot and Schoenfeld residuals. We used Cox's proportional hazards models^15^ to produce hazard ratios (HRs) with accompanying 95% confidence intervals as our estimate of the association between the duration of depressive symptoms and all-cause mortality. Participants with a 0 on the duration of depressive symptoms score were used as the reference group. We compared the mortality rate in this group with four other groups based on the number of waves when they reported elevated depressive symptoms (1, 2, 3 or 4 waves). We examined the ‘shape’ of the association between depressive symptom reporting and mortality by calculating a total of raw CES-D scores across the four waves and dividing people into nine categories (0, 1, 2–3, 4–5, 5–7, 8–10, 11–15, 16–20, >20); again, 0 was the reference group. We also modelled this total as a continuous score, where the hazard ratios represent a one-unit change in the total of CES-D scores across waves. We performed comparisons in survival curves with the log-rank test.

Survival time was measured in months, from the date of interview in the final wave of exposure measurement (2008–2009) to the date of death or 15 March 2012, whichever was first. We assessed whether there were interactions between the duration of depressive symptoms and age and gender in the association with mortality, but found none. We therefore pooled data for men and women and adjusted the hazard ratios for age and gender (the basic model). Hazard ratios were additionally adjusted for socioeconomic position, health behaviours, functional impairments, whether participants lived alone, chronic conditions, circulatory disease, cancer and tests of cognitive function. As depression and cognitive function share symptoms (e.g. impaired concentration, fatigue), we adjusted for all variables both with and without cognitive function. We summarised any changes in hazard ratios after adjusting for each set of covariates using the formula: [HR_basic model_-1]−[HR_adjusted model_-1]/[HR _basic model_-1]×100%.^16^

### Attrition

As longitudinal studies have shown that attrition is more common in participants reporting depressive symptoms in comparison to those who are symptom free,^5^ we conducted a preliminary analysis comparing the duration of depressive symptoms in those who attended at wave 1 with those who attended all four waves. We found that study members who attended all four waves were less likely to report depressive symptoms than those attending on fewer occasions (difference at baseline *P*<0.001). We therefore imputed the data for participants if they answered all items on the CES-D at wave 1; in subsequent waves, if depressive symptoms or covariates were missing, they were imputed.

Data on depressive symptoms and covariates at all waves were available for 5266 people. We imputed missing data from attrition and item non-response using multiple imputation by chained equations which included all variables (including mortality) in the prediction model to generate 10 imputed data-sets (each had a final *n* = 9560).^17^ We ran our analysis in a complete sample of 5266 participants without any missing data across all waves and found essentially the same pattern of results (online Table DS1). Owing to the greater precision offered, we have presented the analyses from the imputed data-sets in this paper.

We conducted a number of sensitivity analyses. To address concerns that somatic complaints brought about by physical disease were a confounding factor, we recalculated totals for CES-D scores across the four waves after removing the three items relating to somatic symptoms. As another cohort of older adults had defined elevated depressive symptoms on the CES-D using a threshold of ⩾4,^18^ we reran our analysis using this threshold. We examined all 16 permutations of duration (e.g. one wave was 1000, 0100, 0010 and 0001) and included deaths that occurred during the assessment of depressive symptoms. Due to the possibility of left-censoring of duration, whereby participants could enter the study having had depression for many years, we excluded participants with depressive symptoms at wave 1. As there is evidence that poor cognitive function predicts drop-out in longitudinal studies with older adults,^19^ we reran the analyses after excluding participants who scored in the lowest quartile on measures of cognitive function. Then, to explore the possibility of reverse causality (whereby imminent death/terminal illness may increase symptoms of depression), we conducted a subgroup analysis by excluding all participants who died within 12 months after the final wave in 2008–2009 (*n* = 250). All analyses were carried out using Stata Version 13.0 (StataCorp LP, College Station Texas).

## Results

Online Fig. DS1 shows how we derived the analytical sample. At wave 1, we interviewed 11 391 men and women.^8^ We excluded proxy interviewees (*n* = 477), those who did not provide a response to all items on the CES-D at wave 1 (*n* = 213) and those who had died between waves 1 and 4 (*n* = 1141), resulting in a cohort of 5266 participants (2922 women) with no missing data and an imputed analytical sample of 9560 (5306 women).

In Table 1, we have shown the baseline characteristics of participants according to the number of waves that they recorded a CES-D score ⩾3. The prevalence of people classified as a case based on the reporting of depressive symptoms on 1, 2, 3 and 4 waves was 19.3% (*n* = 1842), 11.2% (*n* = 1072), 7.9% (*n* = 760) and 6.5% (*n* = 619) respectively. In general, relative to those without depressive symptoms, people who reported a longer duration with depressive symptoms had markedly less favourable characteristics. Study participants reporting a longer duration with symptoms were more likely to be women, older, employed in a routine occupation, be less wealthy, live alone, report a functional impairment, be a current smoker, hardly ever engage in physical activity, not consume alcohol in the past year and report having a health condition (with the exception of cancer).

**Table 1:** Characteristics of participants according to the number of occasions (2002–2003 to 2008–2009) with depressive symptoms (*n* = 9560)

	Number of waves with depressive symptoms						
	None	1	2	3	4	*P*
Number of participants	5267	1842	1072	760	619	

Deaths, % (*n*)	7.4 (390)	10.8 (199)	14.5 (156)	16.2 (123)	19.9 (123)	<0.001

Age, years: mean (s.d.)	62.13 (8.77)	63.08 (9.43)	63.94 (9.69)	64.37 (10.02)	64.63 (10.12)	<0.001

Women, % (*n*)	48.8 (2573)	59.5 (1096)	65.3 (700)	66.6 (506)	69.6 (431)	<0.001

Lowest occupational social class,^a^ % (*n*)	39.1 (2059)	46.7 (860)	52.8 (566)	58.2 (442)	65.4 (405)	<0.001

Lowest quintile in total wealth,^b^ % (*n*)	10.7 (563)	19.0 (350)	23.0 (247)	30.7 (233)	37.9 (235)	<0.001

Living alone, % (*n*)	22.1 (1165)	28.9 (533)	38.2 (409)	43.6 (331)	53.2 (329)	<0.001

Current smoker, % (*n*)	17.1 (769)	22.2 (335)	28.5 (238)	30.4 (177)	41.6 (182)	<0.001

Hardly ever engage in mild physical activity, % (*n*)	6.6 (348)	9.6 (176)	11.8 (127)	17.1 (130)	22.1 (137)	<0.001

No alcohol consumption in past year, % (*n*)	7.1 (376)	11.0 (203)	15.1 (162)	17.5 (133)	22.9 (142)	<0.001

Difficulty in any ADL, % (*n*)	9.1 (481)	18.4 (340)	27.2 (292)	36.5 (277)	51.2 (317)	<0.001

Difficulty in any IADL, % (*n*)	8.1 (427)	17.8 (329)	29.3 (315)	39.6 (301)	55.6 (344)	<0.001

Antidepressant use,^c^ % (*n*)	2.1 (112)	4.9 (90)	7.1 (76)	9.5 (72)	14.5 (90)	<0.001

Diagnoses and health conditions, % (*n*)						
Hypertension	33.4 (1764)	38.0 (700)	42.0 (451)	43.1 (328)	47.9 (297)	<0.001
Angina	6.3 (332)	9.6 (177)	10.3 (111)	13.9 (106)	18.6 (115)	<0.001
Heart attack	4.0 (209)	5.4 (99)	6.2 (66)	7.4 (56)	9.7 (60)	<0.001
Heart condition^d^	8.5 (450)	9.7 (178)	11.7 (125)	13.1 (100)	14.5 (90)	<0.001
Stroke	2.0 (104)	3.4 (63)	4.7 (50)	5.7 (43)	8.4 (52)	<0.001
Diabetes	4.7 (250)	7.6 (140)	8.4 (90)	9.6 (73)	11.6 (72)	<0.001
COPD	3.4 (181)	5.8 (107)	8.1 (87)	10.0 (76)	14.8 (92)	<0.001
Asthma	9.4 (498)	11.4 (210)	13.8 (148)	17.4 (132)	21.8 (135)	<0.001
Arthritis	25.5 (1237)	33.9 (624)	42.2 (452)	46.8 (356)	56.2 (348)	<0.001
Cancer	5.0 (261)	5.4 (99)	5.9 (63)	6.8 (52)	6.1 (38)	0.08

Memory index, mean (s.d.)	17.60 (4.60)	16.64 (4.91)	15.92 (4.76)	15.28 (4.88)	14.64 (5.06)	<0.001

Executive function score, mean (s.d.)	18.41 (3.97)	17.42 (4.13)	16.82 (4.07)	16.15 (4.07)	15.37 (4.05)	<0.001

ADL, activity of daily living; IADL, instrumental activity of daily living; COPD, chronic obstructive pulmonary disorder.

a. Semi-routine and routine occupational social class according to the National Statistics Socio-economic Classification (NS-SEC) classification system.

b. Total wealth excluded debt and regular pension payments.

c. Self-reported use of antidepressants in wave 0 comprised either selective serotonin reuptake inhibitor or tricyclic antidepressant.

d. Heart conditions were murmur, abnormal heart rhythm and congestive heart failure.

### Duration of depression and all-cause mortality

**Fig. 1:**
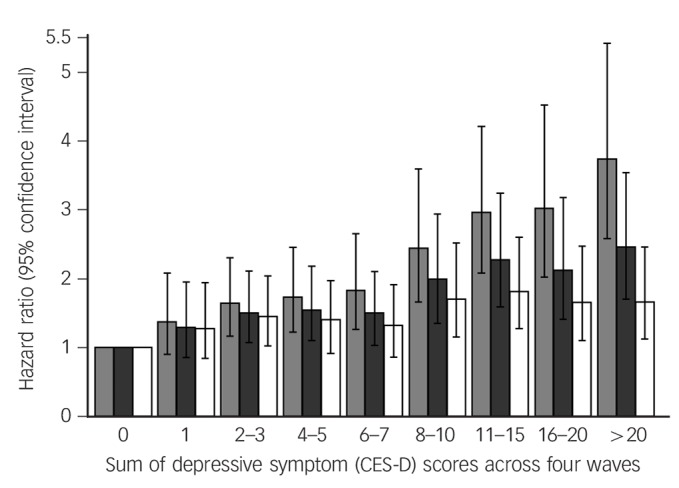
Hazard ratios (95% confidence intervals) for all-cause mortality according to the sum of depressive symptom (CES-D) scores across the four waves (*n* = 9560). Basic model (light grey bars) with health behaviours (smoking status, alcohol consumption (in the past year) and physical activity (dark grey bars); all covariates (white bars). The reference group are participants with a score of zero (category 0). CES-D, Center for Epidemiologic Studies Depression Scale.

There were 991 deaths over an average of 4.2 years of follow-up (s.d. = 0.78) after wave 4 (2008–2009). The age- and gender-adjusted HRs for all-cause mortality among people with depressive symptoms at waves 1, 2, 3 and 4 were 1.41 (95% CI 1.15–1.74), 1.80 (95% CI 1.44–2.26), 1.97 (95% CI 1.57–2.47) and 2.48 (95% CI 1.90–3.23) respectively, compared with people without depressive symptoms (Table 2). The unadjusted survival plots confirmed this dose–response association with the downward slope of survival curves becoming steeper as the number of waves with depressive symptoms increased (log rank = 1624.24; *P*<0.0001) (online Fig. DS2). Figure 1 shows a similar dose–response increase in the risk of mortality, starting at scores of 2–3 (maximum possible score = 32), operating across the full continuum of depressive symptoms. Online Table DS2 shows that when modelled as a continuous measure, a one-unit change in scores was also associated with an increased risk for mortality.

**Table 2:** Hazard ratios (95% confidence intervals) for the association between the number of waves with depressive symptoms (2002–2003 to 2008–2009) and all-cause mortality (*n* = 9560)

	Number of waves with depressive symptoms						
Model	None	1	2	3	4	Per 1 wave increase	*P*
Age and gender (basic model)^a^	1 (ref)	1.41 (1.15–1.74)	1.80 (1.44–2.26)	1.97 (1.57–2.47)	2.48 (1.90–3.23)	1.26 (1.19–1.32)	<0.001

Basic model + socioeconomic position^b^	1	1.36 (1.10–1.68)	1.70 (1.35–2.14)	1.82 (1.44–2.30)	2.20 (1.67–2.89)	1.22 (1.15–1.29)	<0.001

Basic model + living alone	1	1.40 (1.14–1.73)	1.75 (1.40–2.20)	1.89 (1.51–2.38)	2.34 (1.79–3.07)	1.24 (1.18–1.30)	<0.001

Basic model + antidepressant medication^c^	1	1.41 (1.14–1.73)	1.78 (1.42–2.24)	1.93 (1.54–2.44)	2.40 (1.83–3.16)	1.25 (1.18–1.31)	<0.001

Basic model + health behaviours^d^	1	1.26 (1.02–1.55)	1.51 (1.20–1.91)	1.58 (1.24–1.99)	1.74 (1.32–2.30)	1.16 (1.10–1.22)	<0.001

Basic model + any functional impairment^e^	1	1.31 (1.06–1.62)	1.54 (1.22–1.95)	1.58 (1.25–2.00)	1.81 (1.36–2.40)	1.16 (1.10–1.23)	<0.001

Basic model + chronic conditions^f^	1	1.36 (1.11–1.69)	1.71 (1.36–2.15)	1.83 (1.45–2.31)	2.21 (1.67–2.91)	1.22 (1.16–1.29)	<0.001

Basic model + tests of cognitive function^g^	1	1.30 (1.05–1.61)	1.63 (1.29–2.05)	1.70 (1.35–2.13)	2.05 (1.57–2.67)	1.20 (1.14–1.26)	<0.001

Basic model + circulatory disease and risk factors^h^	1	1.37 (1.11–1.69)	1.72 (1.36–2.17)	1.84 (1.46–2.33)	2.18 (1.66–2.85)	1.22 (1.16–1.28)	<0.001

Basic model + all diagnoses and conditions	1	1.33 (1.08–1.65)	1.65 (1.31–2.09)	1.74 (1.37–2.21)	2.00 (1.51–2.64)	1.20 (1.13–1.26)	<0.001

All covariates without cognitive function	1	1.18 (0.95–1.45)	1.31 (1.03–1.67)	1.29 (1.00–1.66)	1.28 (0.95–1.74)	1.07 (1.01–1.14)	0.14

All covariates	1	1.13 (0.91–1.40)	1.26 (0.99–1.61)	1.21 (0.95–1.56)	1.20 (0.89–1.63)	1.06 (0.99–1.12)	0.35

ADL, activity of daily living; IADL, instrumental activity of daily living; COPD, chronic obstructive pulmonary disorder.

a. Basic model is adjusted for age and gender.

b. Socioeconomic position comprised occupational social class according to the National Statistics Socio-economic Classification (NS-SEC) and total wealth (excluding debt and regular pension payments).

c. Self-reported use of antidepressants in wave 0 comprising either selective serotonin reuptake inhibitor or tricyclic antidepressant.

d. Health behaviours comprised smoking status, alcohol intake (per year) and physical activity.

e. Functional impairments were any ADL or IADL.

f. Chronic conditions were COPD, asthma, diabetes (types 1 and 2) and arthritis.

g. Tests of cognitive function were on memory and executive function.

h. Circulatory disease and risk factors comprised hypertension, previous heart attack, stroke, angina and heart conditions.

Adjustment for health behaviours, including current smoking status, alcohol consumption and physical activity reduced the per-wave HR from 1.26 to 1.16 (*P*<0.001 for trend, Table 2), representing a 38.5% attenuation in risk. Adjustment for functional impairments led to the same level of attenuation. Inclusion of measures of baseline health status and tests of cognitive function both reduced the strength of the association by around a quarter (23.1%), with a modest attenuation in HRs observed after adjustment for chronic conditions (15.4%) and circulatory disease (15.4%) (Table 2). After adjusting for all covariates, the association between the number of waves with elevated depressive symptoms and mortality was essentially lost (76.9% reduction in hazard ratios; *P* for trend = 0.35, Table 2).

### Sensitivity analysis

People with missing data were older, had a semi-routine or routine occupation, less total wealth, reported a functional impairment, smoked, were physically inactive and reported an existing heath condition (apart from asthma, cancer and arthritis), but consumed less alcohol (all *P*-values <0.05; results available on request).

We ran the statistical models with a separate series of adjustments for each covariate. Age, moderate intensity physical activity, living alone, measures of memory and executive function, and having any problem with an ADL or IADL yielded the greatest attenuation in hazard ratios (online Table DS3). Models comparing the ⩾3 (Table 2) to ⩾4 threshold on the CES-D used to define elevated depressive symptoms yielded comparable estimates (online Table DS4). Removal of somatic complaints from this total score also essentially had no effect on these estimates (online Table DS5), as did excluding participants with depressive symptoms at wave 1 to remove the possibility of left-censoring (online Table DS6). Hazard ratios were also comparable whether consecutive (e.g. two waves = 1100) or non-consecutive waves (two waves = 1001) with depressive symptoms were used (results available on request), whether participants with poor cognitive function at baseline were or were not excluded (online Table DS7) and when deaths occurring during the assessment of depressive symptoms were included (online Table DS8).

### Testing for reverse causality

To test the reverse causal hypothesis of depressive symptoms increasing before death, we reran the regression models after excluding 250 participants who died within the first 12 months of the follow-up time. This exclusion had little effect on the estimates, with a HR of 1.06 (95% CI 0.99–1.12) in the main analytical sample in comparison to 1.04 (95% CI 0.98–1.12) after excluding deaths in the first 12 months of follow-up (model adjusted for all covariates; online Table DS9).

## Discussion

The main finding of our study was a dose–response association between the duration of depressive symptoms and mortality risk. This graded association was explained largely by differences in engagement in moderate physical activity, cognitive function, functional impairments and physical illnesses.

### Comparison with existing studies

In agreement with results from smaller cohort studies,^3,5–7^ we found that functional impairments and physical illness attenuated the association between the duration of depressive symptoms and mortality, each explaining around a third of the association. In the Established Populations for Epidemiologic Studies of the Elderly project, the largest (*n* = 3701) and most detailed investigation to date, the association between chronic depressive symptoms (defined as screening positive on the CES-D twice over 3 or 6 years) and mortality was fully attenuated after adjustment for differences in health behaviours, health conditions, physical disability and functional impaiments.^7^ In the much smaller, Longitudinal Aging Study Amsterdam study, depressive symptoms lasting 3–3.5 years were associated with mortality after adjustment for functional impairments and physical illnesses, but important covariates such as health behaviours or cognitive function were not accounted for. We replicated the pattern of attenuation to the null after accounting for functional impairments, physical illnesses and cognitive function, but explicitly investigated depressive symptom duration as a count of waves. Our analysis has also extended the results from other cohorts by being a younger cohort at the first assessment of depressive symptoms, with a lower prevalence of chronic disease.^3,6,7^ The present findings suggest that in addition to functional impairments and physical illnesses, physical inactivity and poor cognitive function each explain around a quarter of the association between the duration of depressive symptoms and mortality.

Among the candidate mechanisms linking the duration of depression and mortality, a number of health and lifestyle factors may form part of an indirect mechanism. The attenuation of the association between duration of depression and mortality by physical inactivity and functional impairments we observed suggests these may form part of an indirect mechanism. A recent analysis of the Americans' Changing Lives Study using time-varying assessments provides partial support for this hypothesis by showing that changes in functional impairments and physical illness attenuated the association between baseline depressive symptoms and mortality.^20^ However, two other cohort studies of older adults identified a bidirectional relationship between functional impairments and depressive symptoms,^21,22^ whereas engaging in low levels of physical activity has been shown to slow the progression of mobility impairments,^23^ suggesting that the attenuation by physical activity and mobility impairments may be linked.

A contrasting explanation is that the association between depression and mortality is due to confounding brought about by the effects of physical inactivity, functional impairments and physical illness on depressive symptoms and mortality. Although it is possible that there may be some important unmeasured illnesses or differences in the severity of illnesses we did not account for, adjustment for a wide range of physical illnesses did not completely attenuate the association. Further, the attenuation by cognitive function may be due to some of their shared symptoms and therefore could be an example of overcontrol rather than confounding.

### Strengths and limitations

The strengths of this study are that it is the first to examine the association between the duration of depressive symptoms and mortality in older people. The detailed assessments of socioeconomic status, health behaviours, functional impairments and physical illness, and tests of cognitive function enabled us to examine a wide range of explanatory factors, but some factors (e.g. antidepressant use) were not recorded at each wave and others, such as diet, were not available. In contrast to other studies with smaller sample sizes^4–6^ and short duration of follow-up,^4,6,7^ we were able to examine whether the relationship between mortality and depressive symptoms could have been explained by increases in depressive symptoms with approaching death.^19,24^ We found that hazard ratios for the duration with depressive symptoms and mortality were only slightly reduced when deaths that occurred in the first 12 months were excluded, suggesting that pre-terminal increases in depressive symptoms did not completely account for the association.

The main limitation of this study is loss to follow-up, a perennial problem in longitudinal studies. However, sensitivity analysis comparing data-sets with and without imputed data-sets provided no evidence that missing data introduced bias, increasing confidence in the main findings. In defining covariates, we relied on baseline data rather than data from all four waves because imputation of covariates at subsequent waves would increase uncertainty in multivariable adjusted analyses. Depression symptoms were measured at four discrete assessments during the study period, and there may have been some fluctuation in depression status in between these assessments. Our assessment of the duration of depressive symptoms meant that for some people, duration did not mean consecutive waves with depression. An analysis comparing estimates from consecutive versus non-consecutive waves with depressive symptoms produced comparable estimates, suggesting this did not have a significant impact on estimates.

In conclusion, we found that the risk of mortality increased in a stepwise manner with the duration of depressive symptoms. The progressive nature of this association is unlikely to be explained by reverse cause but may be ascribed to multiple connected pathways involving levels of physical activity, cognitive function, functional impairments and physical illness.
